# Advances in Exercise-Mediated Regulation of the Gut Microbiota via the Muscle–Gut Axis: Implications for Tumor Immunity and Treatment Responses

**DOI:** 10.3390/metabo16090638

**Published:** 2026-09-01

**Authors:** Tao Pang, Xinyi Zhou, Zhe Ge

**Affiliations:** School of Sports, Shenzhen University, Shenzhen 518060, China; 2020128264@email.szu.edu.cn (T.P.); 2020128254@email.szu.edu.cn (X.Z.)

**Keywords:** exercise, muscle–gut axis, gut microbiota, tumor immunity, treatment responses

## Abstract

The gut microbiota can influence tumor immunity and treatment responses through microbial metabolism, intestinal barrier regulation, and immune–inflammatory signaling, but whether exercise engages these mechanisms through the muscle–gut axis remains uncertain. This review integrates evidence from exercise physiology, microbial metabolism, and tumor immunology to examine how exercise-associated host signals may reshape the intestinal ecological niche and microbial function. Three candidate muscle–gut axes are proposed: the myokine–enteroendocrine–substrate delivery–short-chain fatty acid (SCFA) axis, the exercise-associated lactate–microbial cross-feeding–propionate axis, and the muscle-derived endocrine signaling–intestinal epithelial repair–hypoxic niche axis. Separate studies support exercise-associated IL-6/GLP-1/PYY regulation and gastrointestinal transit, lactate entry into the intestinal lumen and lactate-associated microbial remodeling, *Veillonella atypica*-mediated propionate production, and irisin/apelin-related epithelial repair. Together, these pathways may influence microbial metabolism, barrier homeostasis, and immune–tumor interactions. However, tumor-related links remain incomplete, and none has been validated as a complete causal chain in a single tumor-bearing exercise model. Relatively complete preclinical evidence comes from mouse melanoma, where endurance exercise enhanced microbial folate-dependent one-carbon metabolism and formate output, with microbiota-derived formate promoting CD8^+^ T-cell antitumor activity and immune checkpoint inhibitor efficacy. Regular exercise with an appropriate load and adequate recovery may support microbial and intestinal barrier homeostasis, whereas excessive or prolonged exercise with inadequate recovery may impair barrier integrity. Human evidence remains limited and largely associative and does not establish microbiota-mediated improvements in tumor immunity or treatment responses. These candidate axes therefore require causal validation in tumor-bearing exercise models and prospective human studies.

## 1. Introduction

Tumor development, progression, and treatment responses are jointly influenced by multiple factors, including intrinsic characteristics of tumor cells, the host metabolic and inflammatory state, the immune microenvironment, and the microbial ecosystem. The gut microbiota (GM) is one of these important factors [1]. Studies have shown that gut dysbiosis can contribute to tumor progression by promoting chronic low-grade inflammation, disrupting intestinal barrier homeostasis, perturbing host metabolism, and impairing immune surveillance. In contrast, under specific conditions, certain commensal bacteria and their metabolites can enhance dendritic cell (DC) antigen presentation and tumor antigen-specific T-cell responses and improve treatment responses, demonstrating the potential to enhance antitumor immunity [2]. Therefore, the gut microbiota exerts bidirectional and context-dependent effects on tumors, encompassing both tumor-promoting and tumor-suppressive effects. The direction of these effects is jointly determined by the functional state of the microbiota, host background, cancer type, and treatment context [3].

The gut microbiota is highly plastic and can be influenced by factors such as genetics, diet, medications, age, and lifestyle [4]. As a modifiable lifestyle factor, exercise is closely associated with gut microbial ecology, intestinal barrier homeostasis, and the host immune–metabolic state [5]. However, the regulation of the gut microbiota by exercise should not be understood solely in terms of increases or decreases in the abundance of specific genera, but rather within the framework of systemic host adaptation mediated by the muscle–gut axis. Exercise may shape the intestinal ecological niche through endocrine and metabolic communication, improve microbial metabolic function, and maintain microbiota homeostasis, with subsequent intestinal barrier–immune feedback contributing to the regulation of the host inflammatory and immune milieu [6]. Microbiota homeostasis emphasizes a relatively stable and resilient dynamic equilibrium among microbial functions, the intestinal barrier, and mucosal immunity, rather than a fixed microbial composition or the persistent predominance of specific beneficial bacteria [7,8]. This state can be assessed across multiple measurable dimensions, including microbial metabolic function and the output of key metabolites, intestinal epithelial barrier integrity and permeability, microbial molecule translocation (e.g., LPS) and related inflammatory markers, and the extent to which microbial composition and function return toward an individual’s baseline following perturbations such as changes in diet, medications, or exercise load. Therefore, when evaluating exercise-associated changes in the gut microbial ecosystem, greater attention should be paid to microbial function, barrier status, inflammatory burden, and resilience following perturbation, rather than determining whether the microbiota is in a favorable state based on the abundance of a single genus.

At present, direct evidence linking exercise-mediated regulation of the gut microbiota through the muscle–gut axis to tumor immunity and treatment responses remains limited. Existing evidence is derived mainly from animal models and a limited number of cancer types, whereas human studies are largely correlational or provide indirect evidence, making it difficult to exclude confounding factors such as diet, medications, body fat levels, and treatment stage. Against this background, existing reviews have primarily focused on exercise-associated changes in gut microbiota composition or associations between the microbiota and tumor immunity, with relatively little integration of the continuous process encompassing exercise-induced host adaptation, changes in the intestinal ecological niche, remodeling of microbial function, and immune feedback. From the perspective of the muscle–gut axis, this review integrates exercise-induced host signals, changes in the intestinal ecological niche, remodeling of microbial function, and immune feedback, with particular emphasis on evidence for microbiota-mediated exercise effects, the completeness of evidence supporting different mechanistic chains, and their limits of applicability. Following the sequence of “exercise stimulus–host ecological niche alteration–microbial functional remodeling–intestinal barrier and immune feedback–tumor-related implications,” this review provides a conceptual framework for future studies evaluating how gut microbiota-related mechanisms may inform stratified and individualized exercise strategies for cancer prevention and treatment. The muscle–gut axis framework proposed in this review is based primarily on existing evidence from different levels and is intended to summarize potential regulatory pathways, rather than to imply that a complete causal chain whereby exercise improves tumor immunity through the gut microbiota has already been established.

PubMed, Web of Science, and Scopus were searched from database inception through 31 July 2026. Search terms covered exercise/physical activity, skeletal muscle/myokines/muscle–gut axis, gut microbiota/microbial metabolites, intestinal barrier, and tumor immunity/treatment responses, supplemented by reference tracking. Peer-reviewed original studies were used as the primary evidence, with emphasis on studies examining exercise-related changes in the gut microbiota or microbial metabolites, host signaling and regulation of the intestinal ecological niche, and associations of the microbiota/metabolites with tumor immunity, tumor progression, or treatment responses. Representative reviews were used only for mechanistic background and conceptual definitions, whereas studies with limited relevance to the review topic or insufficient key information were not considered core evidence. Given the heterogeneity of study models and causal pathways, studies were first categorized as in vitro, animal mechanistic, human observational, or human interventional studies, and were then qualitatively evaluated according to the directness of the evidence and the degree of causal support. Particular attention was given to whether the relevant components were evaluated within the same experimental framework and whether causal validation was performed using fecal microbiota transplantation, microbiota depletion, or key metabolite interventions. These criteria were subsequently applied to the synthesis of representative exercise studies and the definition of their corresponding interpretive boundaries.

## 2. Mechanistic Basis of the Effects of the Gut Microbiota on Tumor Immunity and Treatment Responses

The gut microbiota can contribute to the development and progression of various cancers, including colorectal cancer, pancreatic cancer, and hepatocellular carcinoma [9,10,11], and interindividual differences in microbiota composition and related functional characteristics are also associated with differences in responses to cancer immunotherapy [12,13]. The gut microbiota can exert bidirectional effects on tumor progression, either promoting or suppressing it, through the production and transformation of microbial metabolites, regulation of innate and adaptive immune responses, and remodeling of the tumor microenvironment [13]. For tumors located within the intestine, certain luminal or mucosa-associated microorganisms and their metabolites can act directly on the intestinal mucosa and tumor tissue [9,14]. For distant extraintestinal tumors, microbial metabolites can be absorbed across the intestinal epithelium into the portal vein and subsequently undergo hepatic uptake and metabolism. Parent compounds that are not completely taken up by the liver, together with some transformed products, can further enter the systemic circulation, reach distant tissues, and either act directly on local cells or contribute to shaping the systemic immune–metabolic milieu.

Meanwhile, gut-derived microbial antigens can induce immune responses in gut-associated lymphoid tissues and mesenteric lymph nodes, and the resulting immune mediators, as well as effector or memory immune cells that enter the circulation, can contribute to systemic immune regulation [13,15,16]. However, these processes primarily reflect the shaping of systemic immune status by gut-derived signals and are not equivalent to the initial priming of tumor antigen-specific T cells, which occurs mainly in tumor-draining lymph nodes. Activated T cells subsequently enter the tumor microenvironment through the lymphatic and blood circulation [17]. When intestinal barrier permeability is increased, lipopolysaccharide (LPS) and other microbe-associated molecules are more likely to translocate and enter the portal vein. Those not completely cleared by the liver can further enter the systemic circulation and activate TLR-associated pro-inflammatory signaling. At the same time, gut dysbiosis can also promote the accumulation of myeloid-derived suppressor cells, thereby creating an unfavorable tumor immune environment [15,18]. Therefore, elucidating the metabolic, barrier, and immune communication mechanisms associated with the gut microbiota not only contributes to understanding the microbial ecological basis of cancer progression but also provides a theoretical basis for further investigating whether exercise can participate in tumor immunity and treatment responses through remodeling of the gut microbiota.

### 2.1. Gut Microbiota-Associated Metabolites and the Regulation of Tumor Immunity

Short-chain fatty acids (SCFAs) produced through gut microbial metabolism are among the more extensively studied microbial metabolites [19]. Among them, butyrate is a representative SCFA that has been relatively well studied, and its production is commonly associated with butyrate-producing bacteria such as *Faecalibacterium prausnitzii* and members of the genera *Roseburia*, *Eubacterium*, and *Coprococcus*. Butyrate can inhibit tumor cell growth by suppressing histone deacetylase (HDAC) activity, inhibiting the expression of inflammation- and proliferation-related genes, and promoting apoptosis- and cell-cycle arrest-related programs. These effects have been demonstrated in models of colorectal cancer, lymphoma, and breast cancer [20,21,22,23]. In colorectal cancer, butyrate can downregulate the oncogene c-Myc and c-Myc-induced transcription of the miR-17-92a cluster, thereby relieving its suppression of the cell-cycle inhibitor p57 and consequently inhibiting cancer cell proliferation and inducing apoptosis [24]. In addition, butyrate can act as a ligand for GPR109A, promoting anti-inflammatory characteristics in colonic mucosal macrophages and DCs and promoting Foxp3^+^ regulatory T cells (Tregs) and IL-10^+^ CD4^+^ T-cell responses, thereby suppressing colonic inflammation and reducing the risk of inflammation-associated colorectal cancer [25,26]. At the level of T-cell immunity, butyrate can induce the expression of the T helper 1 (Th1)-associated transcription factor T-bet and the effector molecule interferon-γ (IFN-γ) in CD4^+^ T cells. Butyrate and valerate can also enhance the expression of effector molecules and the antitumor activity of CD8^+^ T cells and chimeric antigen receptor T cells (CAR-T cells), suggesting that some SCFAs may have the potential to link microbial metabolism with antitumor T-cell immunity [27,28].

The gut microbiota can also produce a variety of tryptophan metabolites. For example, *Lactobacillus*-derived indole-3-lactic acid (ILA) demonstrated antitumor activity in both mouse models and patient-derived colorectal cancer organoids [29]. Its mechanisms include enhancing the antitumor function of CD8^+^ T cells [30] and inhibiting T helper 17 (Th17) cell differentiation and downregulating the IL-17 signaling pathway by targeting RAR-related orphan receptor γt (RORγt) [31]. In addition, ILA generated through the catabolism of L-tryptophan by *Bifidobacterium breve* can promote the differentiation of immature inflammatory colonic macrophages into mature homeostatic macrophages, thereby improving the precancerous inflammatory intestinal milieu and suppressing tumorigenesis [32]. Another tryptophan metabolite, indole-3-acetic acid (IAA), can upregulate the expression of interferon-γ-inducible protein 10 (C-X-C motif chemokine ligand 10, CXCL10), promote the recruitment and infiltration of CD8^+^ T cells into tumor tissues, and enhance the sensitivity of mice with colorectal cancer and melanoma to anti-programmed cell death protein 1 (PD-1) therapy [33]. Thus, tryptophan metabolites not only participate in the regulation of local intestinal inflammatory homeostasis but can also influence tumor progression and treatment responses through immune-related mechanisms in certain tumor models.

**Figure 1:**
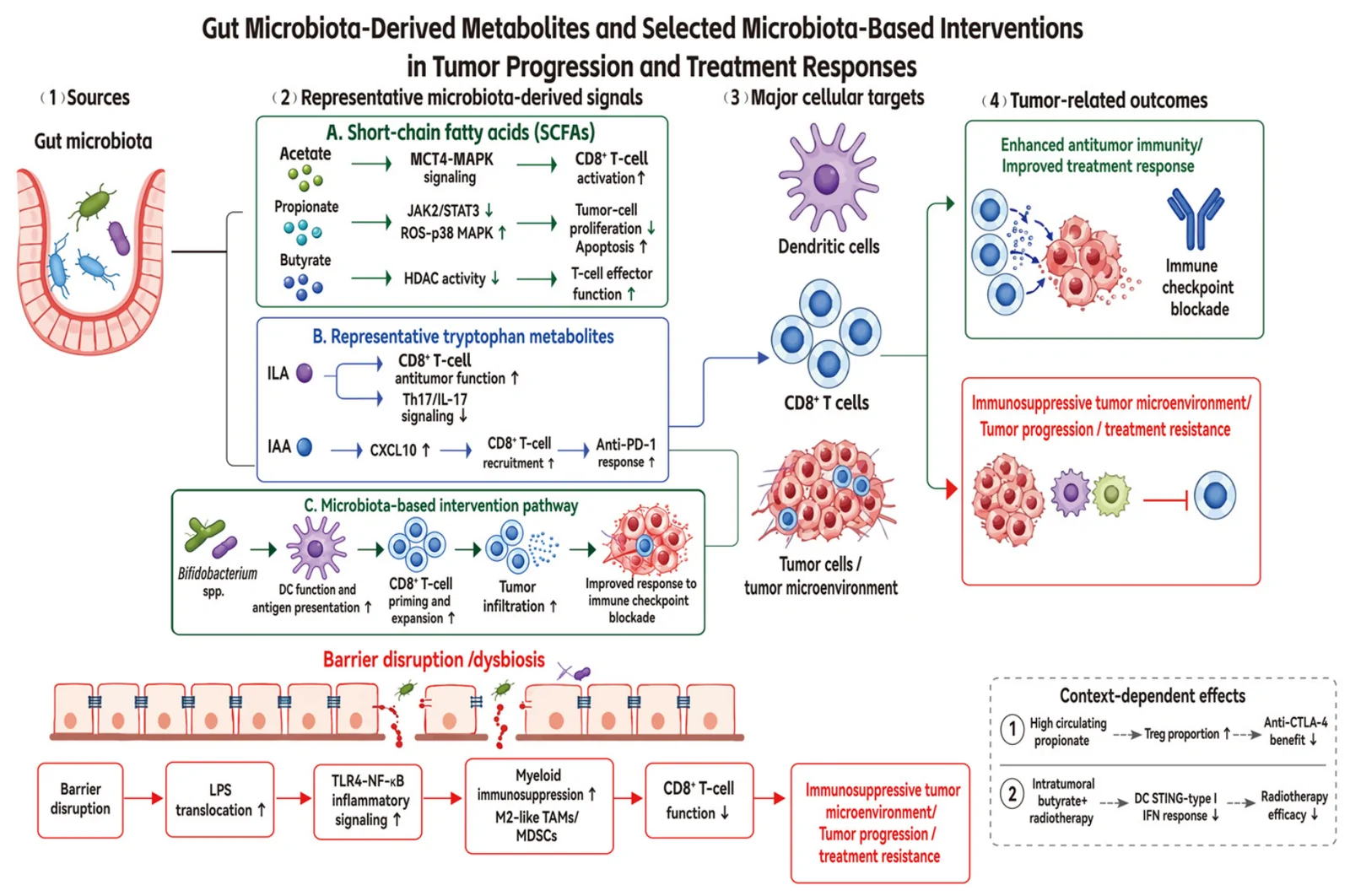
Schematic overview of gut microbiota-derived metabolites and microbiota-associated mechanisms involved in tumor progression and treatment responses. Microbial metabolites, microbial molecules, and microbiota-based interventions can influence tumor cells, immune responses, intestinal barrier function, and treatment responses. The lower red pathway illustrates barrier disruption/dysbiosis-associated inflammatory and immunosuppressive effects. Green and red arrows or boxes indicate predominantly antitumor or immune-enhancing and tumor-promoting or immunosuppressive effects, respectively; the gray dashed box indicates context-dependent effects. Arrows denote process sequences or directions of effects. ↑ indicates increase or enhancement; ↓ indicates decrease or inhibition.

In addition, microbiota-associated metabolites/molecules, including bile acids, lactate, LPS, and inosine, can influence tumor progression and treatment responses by regulating intestinal barrier function, inflammatory signaling, immune cell states, and tumor metabolic adaptation. Although LPS is not a metabolite, it can serve as an important signal of intestinal barrier disruption and the translocation of Gram-negative bacterial components and can participate in inflammatory amplification and the formation of an immunosuppressive microenvironment through Toll-like receptor 4 (TLR4)-related pathways [11,34,35,36]. The biological effects of microbial metabolites are not fixed but depend on their source, the context in which they act, and the disease setting. Therefore, findings observed in different cancer types or treatment settings cannot simply be extrapolated from one context to another. For example, propionate can directly inhibit tumor cell growth in breast cancer and colorectal cancer models [37,38], whereas higher circulating propionate levels are associated with an increased proportion of Tregs and reduced benefit from anti-cytotoxic T-lymphocyte-associated protein 4 (CTLA-4) therapy [39]. Butyrate can support mucosal homeostasis and T-cell effector function [25,26,27,28]; however, in the context of radiotherapy, local butyrate within tumors can suppress the STING–type I IFN response in DCs and reduce the efficacy of radiotherapy [40]. When relating these findings to exercise studies, the microbial source of the metabolite, exercise-associated changes, and tumor-related effects should be considered separately, and the effects of metabolites observed in independent cancer models should not be directly regarded as microbiota-mediated mechanisms of exercise effects. An overview of the mechanisms operating in these different contexts is presented in Figure 1, while the biological sources, principal sites of action, and cancer/model evidence for individual metabolites/molecules are summarized in Table 1.

### 2.2. Regulation of Innate Antitumor Immunity by the Gut Microbiota

The gut microbiota can regulate innate immune cell function through microbial components, metabolites, and pattern-recognition receptor signaling, thereby influencing tumor immune surveillance and the state of the tumor microenvironment (TME). This regulation does not depend solely on changes in the abundance of a particular microbial genus but reflects the integrated effects of microbiota-associated signals on immune effector processes involving DCs, macrophages, myeloid-derived suppressor cells (MDSCs), natural killer (NK) cells, and other immune cells. Under conditions of microbiota homeostasis or intervention with specific commensal bacteria, microbiota-associated signals can support DC-mediated antigen presentation and antigen-specific priming of CD8^+^ T cells in tumor-draining lymph nodes, thereby enhancing antitumor immunity [17,73]. In mouse melanoma models, oral administration of *Bifidobacterium* can enhance DC function, promote the priming and expansion of CD8^+^ T cells in tumor-draining lymph nodes, and increase their accumulation in the TME, thereby producing synergistic antitumor effects with programmed death-ligand 1 (PD-L1) blockade [73]. Another study showed that microbiota-derived STING agonists can induce intratumoral monocytes to produce type I interferons (type I IFNs), remodel the composition and functional states of tumor-infiltrating mononuclear phagocytes, increase immunostimulatory monocytes and DCs, and enhance NK–DC interactions within the TME, thereby improving the efficacy of immune checkpoint blockade (ICB) [74]. These findings indicate that microbiota-associated signals can support the DC–CD8^+^ T-cell axis in tumor-draining lymph nodes and remodel mononuclear phagocyte populations and NK–DC functional networks within the TME. The effects of SCFAs and indole metabolites on mucosal inflammatory homeostasis and myeloid cell function are summarized in Table 1.

In contrast, gut dysbiosis can chronically activate inflammatory signaling and drive the TME toward a state of myeloid immunosuppression. In a colorectal cancer model, dysbiosis simulated by oral gavage with *Escherichia coli* was accompanied by an increased LPS burden and overexpression of cathepsin K (CTSK) in tumor cells. Tumor-derived CTSK can bind to TLR4 on tumor-associated macrophages and induce M2 polarization through an mTOR-dependent pathway, further promoting IL-10 and IL-17 release and enhancing nuclear factor-κB (NF-κB)-associated signaling involved in invasion and metastasis [34]. MDSCs represent another key mechanism through which gut dysbiosis impairs antitumor immunity. For example, *Peptostreptococcus anaerobius* can induce MDSC infiltration and activation, suppress the function of effector T cells, particularly CD8^+^ cytotoxic T cells, and mediate resistance to anti-PD-1 therapy in colorectal cancer [18]. In addition, studies of cholangiocarcinoma suggest that TLR4 activation can promote MDSC recruitment through CXCL1 and generate an immunosuppressive microenvironment [75], while studies of *Candida tropicalis* have shown that it can promote MDSC expansion or activation through NLRP3/IL-1β signaling [76]. These findings indicate that dysbiosis associated with bacteria and fungi from different sources may contribute to tumor progression through MDSC-mediated myeloid immunosuppression. Thus, gut dysbiosis not only manifests as alterations in microbiota composition but can also impair tumor immune surveillance through inflammatory activation, immunosuppressive remodeling of myeloid cells, and restriction of effector T-cell function.

The state of the microbiota can also affect innate or innate-like immune effector processes involving neutrophils, NK cells, and γδ T cells, with the direction of these effects depending on strain type, tumor type, and treatment context. Studies have shown that, following antibiotic-induced disruption of the commensal gut microbiota or under germ-free conditions, oxaliplatin-induced reactive oxygen species production and cytotoxicity in tumor-infiltrating myeloid cells are reduced, accompanied by diminished efficacy of platinum-based chemotherapy, suggesting that an intact commensal microbiota helps maintain treatment-associated myeloid cell effector functions [77]. In mouse tumor models treated with cyclophosphamide, *Enterococcus hirae* and *Barnesiella intestinihominis* can also participate in treatment-induced immunomodulation. *E. hirae* is associated with an increased intratumoral cytotoxic T lymphocyte (CTL)/Treg ratio in tumor-bearing mice, whereas *B. intestinihominis* is associated with increased infiltration of IFN-γ^+^ γδ T cells [78]. In contrast, the Fap2 surface protein of *Fusobacterium nucleatum* can bind to the inhibitory receptor T-cell immunoreceptor with Ig and ITIM domains (TIGIT) on NK cells, impair NK-cell cytotoxicity, and promote tumor immune escape [79]. Overall, the effects of the gut microbiota on innate antitumor immunity are bidirectional and context-dependent. Microbiota homeostasis or signals associated with specific commensal bacteria can support antigen presentation by DCs in tumor-draining lymph nodes and remodel the composition and functional states of mononuclear phagocytes within the TME. They can also lead to a relative increase in immunostimulatory monocytes and DCs and enhance NK–DC interactions. In contrast, gut dysbiosis can be accompanied by an increased LPS burden and enhanced TLR4-associated inflammatory signaling, promote the recruitment, expansion, or activation of MDSCs within the TME, and suppress the cytotoxic function of tumor-infiltrating NK cells, thereby driving the TME toward an immunosuppressive state.

### 2.3. Regulation of Adaptive Antitumor Immunity by the Gut Microbiota

The gut microbiota and the associated microbial antigen repertoire can participate in adaptive antitumor immunity through antigen cross-reactivity, the DC–T-cell axis, and metabolite signaling. Some tumor neoantigens share sequence or structural similarities with microbial epitopes and can induce cross-reactive T-cell responses, a mechanism known as “molecular mimicry.” In long-term survivors of pancreatic cancer, T-cell responses to high-quality neoantigens and MUC16-derived neoantigens have been detected both within tumors and among circulating T cells in peripheral blood. Some of these T-cell clones can also recognize microbial epitopes predicted to be cross-reactive, suggesting that certain microbial epitopes may cross-react with tumor neoantigens through molecular mimicry. Studies demonstrating cross-reactivity between an enterococcal bacteriophage-derived antigen and tumor major histocompatibility complex class I (MHC-I)-restricted antigens further indicate that the microbial antigen repertoire may provide antigenic sources for the generation or expansion of cross-reactive T-cell clones, thereby contributing to the shaping of the antitumor T-cell recognition repertoire [80,81].

The DC–T-cell axis is an important component through which the gut microbiota participates in tumor antigen-specific T-cell responses. Tumor-derived antigens can be processed by conventional type 1 dendritic cells (cDC1s) and presented in tumor-draining lymph nodes to prime CD4^+^ and CD8^+^ T cells. After being licensed by CD4^+^ T cells, cDC1s can further enhance CD8^+^ T-cell responses [17]. Activated tumor antigen-specific CD8^+^ T cells subsequently enter the tumor microenvironment through the lymphatic and blood circulation and exert cytotoxic effects [13,17]. During immune checkpoint blockade therapy, specific gut commensal bacteria or defined microbial consortia can enhance antitumor immune responses by promoting DC maturation, inducing Th1-type responses, and enhancing IFN-γ^+^ CD8^+^ T-cell responses. Studies of CTLA-4 blockade have shown that specific members of the genus *Bacteroides*, represented by *Bacteroides fragilis*, can induce IL-12-dependent Th1-type responses and promote DC maturation, thereby enhancing therapeutic efficacy [82]. A defined consortium consisting of 11 human commensal strains can induce IFN-γ^+^ CD8^+^ T-cell responses in the colonic lamina propria in a manner dependent on CD103^+^ DCs and MHC-Ia and enhance the response of extraintestinal syngeneic tumors to immune checkpoint inhibitors [83], suggesting an association between local intestinal immune activation and enhanced responses of distant tumors. Clinical studies further suggest that responses to anti-PD-1 therapy in patients with metastatic melanoma are associated with gut microbiota composition, microbial diversity, and T-cell-related immune characteristics. Although the microbial taxa associated with treatment efficacy are not entirely consistent across cohorts, these findings collectively support an influence of microbiota status on responses to immune checkpoint inhibitors (ICIs) [12,84]. In addition to antigen presentation, microbial metabolites such as butyrate, valerate, indole-3-lactic acid, and indole-3-acetic acid can also regulate T-cell differentiation and effector function, as summarized in Table 1. The regulation of Tregs by the microbiota is strongly context-dependent. Tregs contribute to the maintenance of intestinal mucosal immune tolerance, whereas abnormal enrichment of Tregs within the TME can suppress effector T-cell responses and is associated with poor prognosis in multiple cancers [85]. In patients with metastatic melanoma treated with ipilimumab, baseline enrichment of *Faecalibacterium* and other members of the Firmicutes was associated with clinical benefit but was also accompanied by a higher risk of immune-related colitis, suggesting that clinical benefit and immune-related toxicity may share, in part, a common basis of immune activation [86].

**Figure 2:**
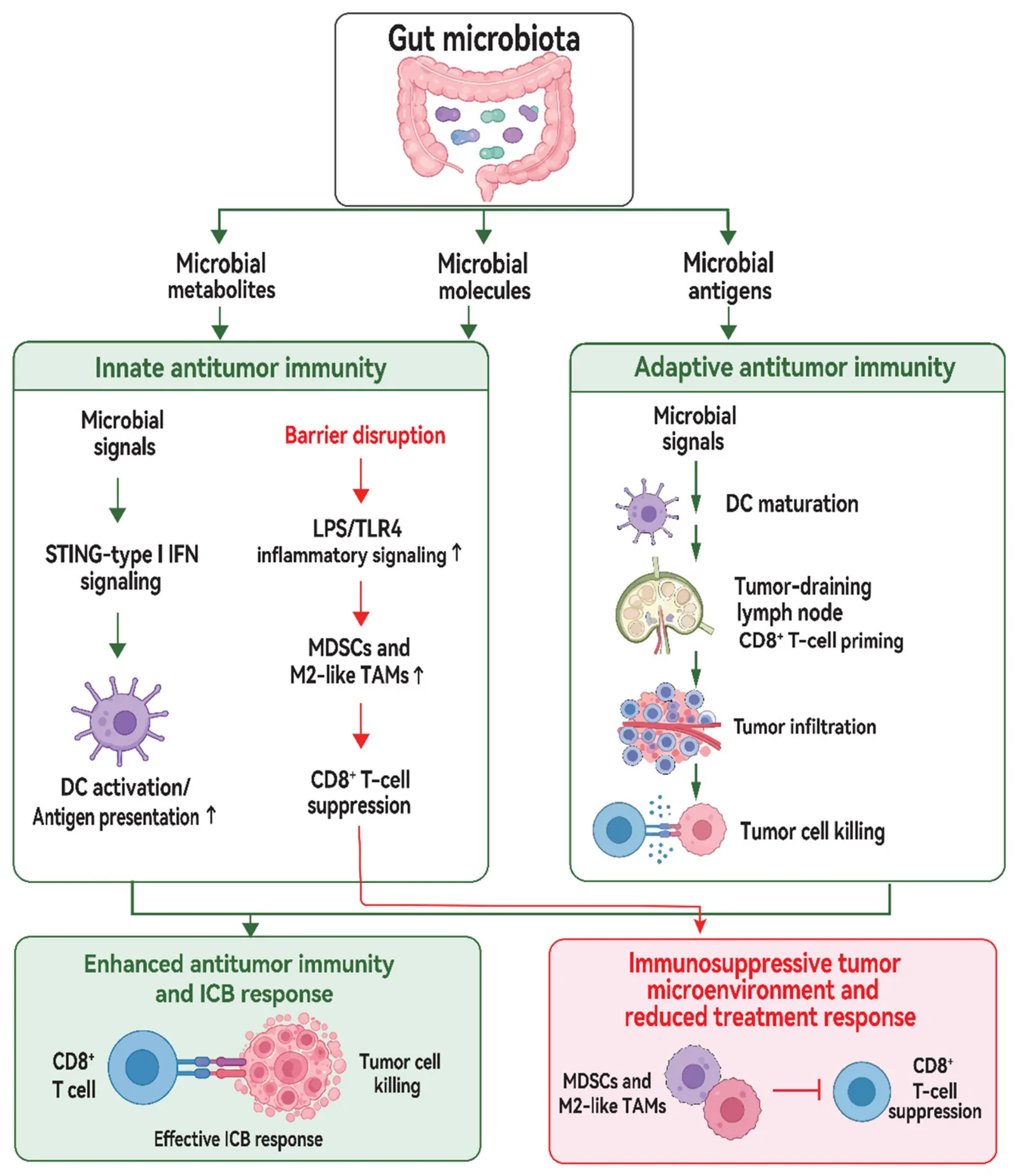
Schematic overview of gut microbiota-mediated regulation of innate and adaptive antitumor immunity and treatment responses. Microbiota-derived metabolites, microbial molecules, and microbial antigens can modulate distinct innate and adaptive immune pathways, including dendritic cell activation and antigen presentation, CD8^+^ T-cell priming and tumor infiltration, and barrier disruption-associated inflammatory and myeloid immunosuppressive signaling. Green and red arrows or boxes indicate predominantly antitumor or immune-enhancing and inflammatory, immunosuppressive, or tumor-promoting effects, respectively. Arrows denote process sequences, cellular trafficking, or directions of effects; blunt-ended lines indicate inhibition. ↑ indicates increase or enhancement; ↓ indicates decrease or inhibition.

Direct evidence that the gut microbiota influences tumor immunity through B-cell-mediated pathways remains limited. Previous studies have shown that the gut microbiota and its TLR2/9 ligands can induce colonic IL-10^+^ regulatory B cells and, through TLR2–MyD88–PI3K-associated signaling, limit mucosal inflammation and maintain colonic immune homeostasis [87]. In the tumor context, the effects of such regulatory responses may be context-dependent. Studies in models of colitis-associated cancer further indicate that B-cell-mediated regulatory immunity and IgA plasma-cell responses can limit tumor-promoting intestinal inflammation [88]. However, their roles in other cancer types and tumor microenvironments remain unclear, and these mechanisms are currently more appropriately considered potential links between mucosal immunity and tumorigenesis. The major mechanisms through which the gut microbiota regulates adaptive antitumor immunity are summarized in Figure 2.

## 3. Exercise-Mediated Regulation of the Gut Microbiota and Its Immune-Related Effects

The effects of exercise on gastrointestinal homeostasis and the gut microbiota cannot be classified solely according to exercise intensity; rather, they depend on whether exercise intensity, duration, total training load, recovery status, heat stress, energy availability, and the individual’s baseline status collectively remain within a tolerable adaptive window. Regular exercise with an appropriate training load and adequate recovery is generally associated with more stable microbial metabolic function and intestinal barrier function, as well as lower intestinal permeability and inflammatory burden [89]. High-intensity interval training (HIIT) and sprint interval training (SIT) with a controlled total load, appropriate interval structure, and adequate recovery should not be equated with ultra-endurance exercise, prolonged continuous strenuous exercise, or high-load exercise with insufficient recovery. The latter, particularly when accompanied by heat stress, dehydration, or low energy availability, is more likely to increase the risk of intestinal hypoperfusion, barrier dysfunction with increased intestinal permeability, and gut dysbiosis [90,91]. Therefore, the effects of exercise on the gut microbial ecosystem should be evaluated in the context of overall exercise load and individual tolerance rather than being determined by exercise intensity alone [92]. To facilitate comparisons across studies, this review follows the exercise prescription definitions used in the original studies for endurance exercise, moderate-intensity continuous training (MICT) and moderate-intensity interval training (MIIT), HIIT, and SIT. The terms “strenuous exercise” and “high-load exercise” are used primarily to describe stressful exposures characterized by prolonged duration, high total training load, or inadequate recovery and are not used synonymously with prescribed HIIT/SIT. 

### 3.1. Mechanistic Basis of Muscle–Gut Axis-Mediated Regulation of the Gut Microbiota by Exercise

From the broader perspective of the muscle–gut axis, the effects of exercise on the gut microbiota are not limited to increases or decreases in the abundance of specific microbial taxa, but instead represent a continuous process encompassing “exercise stimulus–release of skeletal muscle-derived and systemic host signals–alteration of the intestinal ecological niche–microbial functional responses–barrier/immune feedback” [93,94]. Based on the available evidence from different experimental systems, this review proposes three candidate muscle–gut axes: the myokine–enteroendocrine–substrate delivery–SCFA axis, the exercise-associated lactate–microbial cross-feeding–propionate axis, and the muscle-derived endocrine signaling–intestinal epithelial repair–hypoxic niche axis. These three candidate pathways provide a framework for considering how exercise-associated host signals may influence microbial metabolic networks through three interfaces: fermentable substrate delivery, microbial utilization of exercise-associated metabolites, and intestinal epithelial repair with maintenance of the hypoxic colonic niche. These pathways are constructed from evidence supporting individual components across different studies, and none has yet been causally validated across the entire pathway within a single tumor-bearing exercise model.

Skeletal muscle-derived endocrine signals represent an important upstream link through which exercise connects enteroendocrine function with microbial metabolism. Interleukin-6 (IL-6) is a myokine whose release during exercise is well established, and its levels are influenced by exercise load, duration, and pre-exercise muscle glycogen status [95]. In contrast to the sustained elevation of IL-6 under chronic inflammatory conditions, exercise-induced IL-6 elevations are generally transient and pulsatile and primarily contribute to substrate mobilization and interorgan metabolic communication. The “skeletal muscle contraction–IL-6–GLP-1” pathway is a representative route through which muscle contraction influences the enteroendocrine system. Studies have shown that IL-6 can stimulate glucagon-like peptide-1 (GLP-1) secretion from intestinal L cells and pancreatic α cells [96,97]. GLP-1 can delay gastric emptying, enhance satiety signaling, and promote glucose-dependent insulin secretion [98]. Peptide YY (PYY), another postprandial satiety peptide secreted by intestinal L cells, suppresses appetite and delays gastric emptying and small-intestinal transit [99,100]. Together, GLP-1 and postprandial PYY signaling enhance satiety and delay gastric emptying and small-intestinal transit, resulting in a more gradual delivery of postprandial substrates to the distal intestine. When the diet provides adequate fermentable substrates, enhanced satiety signaling may help limit excessive intake at a single meal and reduce abrupt fluctuations in postprandial substrate delivery to the distal intestine. If sufficient dietary fiber and resistant starch reach the colon, this more gradual delivery may support a steadier supply of fermentable carbohydrates.

Once these carbohydrates reach the colon, they are sequentially utilized by microbial groups with different functional capacities. Carbohydrate-fermenting microbial communities comprise groups classified according to their carbohydrate-utilization functions, including primary degraders that break down complex polysaccharides and secondary consumers that further utilize oligosaccharides, monosaccharides, and fermentation intermediates. These functionally distinct members form a continuous carbohydrate-fermentation network through sequential substrate utilization and cross-feeding. Some members of the genus *Bacteroides* can degrade complex polysaccharides in dietary fiber into oligosaccharides and monosaccharides [101], whereas starch-degrading bacteria such as *Ruminococcus bromii* participate in the primary degradation of resistant starch and provide degradation products that can subsequently be utilized by other bacteria [102]. Some members of the genus *Bifidobacterium* can further utilize these carbohydrates to produce acetate and lactate, providing fermentable substrates that can subsequently be utilized by certain butyrate-producing bacteria [103]. In addition to cross-feeding mediated by acetate and lactate, succinate is another important fermentation intermediate exchanged among gut microbes. Some *Bacteroides* species can produce propionate through the succinate pathway, while succinate-utilizing bacteria represented by the genus *Phascolarctobacterium* can take up succinate produced by other gut bacteria and further convert it into propionate [47,104,105]. Meanwhile, members of the genus *Faecalibacterium*, represented by *Faecalibacterium prausnitzii*, as well as *Roseburia*, certain members of *Eubacterium*, and *Coprococcus*, can produce butyrate from carbohydrates or intermediate metabolites generated by other bacteria and are important microbial groups associated with colonic butyrate production [48,106]. Thus, functionally distinct members of carbohydrate-fermenting microbial communities can progressively convert fermentable carbon sources into SCFAs such as acetate, propionate, and butyrate through sequential substrate utilization and metabolite cross-feeding, thereby increasing the overall potential for SCFA production.

As discussed above, SCFAs can support intercellular tight junctions and the mucus barrier of the intestinal epithelium. For example, butyrate can enhance epithelial barrier function by activating AMP-activated protein kinase (AMPK) and promoting tight-junction assembly [107]. Animal studies have further shown that butyrate-associated improvements in barrier function are accompanied by reduced circulating LPS levels and attenuation of low-grade inflammation [108]. However, a reduction in overall food intake does not necessarily benefit the microbiota, particularly if fermentable carbohydrate intake becomes insufficient. Mouse studies have shown that when dietary fermentable carbohydrates are insufficient, mucin-utilizing bacteria such as *Akkermansia muciniphila* and *Bacteroides caccae* can increase their utilization of host mucin glycans. If mucus degradation outpaces host replenishment, the colonic mucus layer may become thinner, increasing the risk of pathogen access to the intestinal epithelium, colonization, and infection [109]. At the same time, insufficient fermentable carbohydrates limit carbohydrate fermentation and SCFA production. The accompanying barrier impairment and increased inflammation can increase the luminal availability of respiratory electron acceptors such as oxygen and nitrate, further compromising the niche of obligately anaerobic SCFA-producing bacteria and promoting the expansion of facultatively anaerobic bacteria, particularly members of the Enterobacteriaceae [110,111,112]. In addition, propionate can stimulate GLP-1 and PYY release from enteroendocrine L cells through free fatty acid receptor 2 (FFAR2), thereby providing feedback from SCFAs to satiety and gastrointestinal motility signaling [113,114]. Existing studies independently support exercise-associated IL-6/GLP-1/PYY regulation, gastrointestinal transit, microbial carbohydrate fermentation, and the barrier-related effects of SCFAs; however, a continuous link from exercise-induced changes in substrate delivery to microbial cross-feeding and SCFA output has not yet been validated.

Exercise-induced metabolites provide another interface for host–microbial cross-feeding. During exercise, skeletal muscle glycolysis increases and is accompanied by elevated circulating lactate. A ^13^C_3_-lactate tracing study in mice showed that systemic lactate can cross the intestinal epithelium and enter the cecal and colonic lumen, thereby providing a potential substrate for gut microbes. However, the experiment used exogenous intravenous lactate, and no labeled propionate was detected during the short-term tracing period. It therefore cannot quantify the contribution of skeletal muscle-derived lactate during exercise to the luminal lactate pool or determine how much of this lactate is subsequently converted to propionate by the microbiota [115]. A 6-week HIIT study in mice provided complementary evidence supporting a role for lactate in exercise-associated gut microbiota remodeling: exogenous lactate partially reproduced HIIT-associated changes in microbial composition and predicted functional features, whereas inhibition of monocarboxylate transporters 1 and 2 (MCT1/2) attenuated some HIIT-induced microbial changes [116]. In humans, the abundance of *Veillonella* increased after marathon running, and subsequent strain-level and experimental studies demonstrated that *Veillonella atypica* can utilize lactate to produce propionate [115]. Collectively, these studies provide component-level experimental evidence that systemic lactate can enter the intestinal lumen, that lactate may participate in exercise-associated microbiota remodeling, and that *V. atypica* can utilize lactate to produce propionate, thereby supporting a cross-feeding link between exercise-associated lactate and microbial metabolism. However, the in vivo contribution of exercise-derived lactate to propionate production through this pathway remains unclear.

Independent of these exercise studies, cancer models have shown that propionate can induce G_0_/G_1_ cell-cycle arrest, inhibit proliferation, and promote apoptosis in breast cancer models by suppressing JAK2/STAT3 signaling and activating reactive oxygen species (ROS)–p38 mitogen-activated protein kinase (MAPK) signaling [37]. In colorectal cancer models, propionate can also upregulate HECTD2, promote the proteasomal degradation of EHMT2, and relieve its epigenetic repression of TNFAIP1, thereby inhibiting tumor cell growth and promoting apoptosis [38]. The effects of propionate also depend on dose, tissue site, and immune context. Higher circulating propionate levels are associated with an increased proportion of Tregs and reduced benefit from anti-CTLA-4 therapy [39], and propionate can also suppress IL-12 production by antigen-presenting cells and impair antigen-specific CD8^+^ T-cell activation [117]. In addition, exercise may provide a complementary pathway by remodeling microbial tryptophan metabolism. A running intervention study observed remodeling of gut microbiota composition and tryptophan metabolic pathways [118]. Different members of *Lactobacillus*, *Bifidobacterium*, and certain Clostridia-related microbial groups can convert tryptophan into derivatives including indole-3-lactic acid, indole-3-acetic acid, indole-3-propionic acid, and indole-3-aldehyde [29,33,55,60]. Among these, aryl hydrocarbon receptor (AhR) ligands such as indole-3-aldehyde can enhance epithelial antimicrobial defense and mucosal immune homeostasis [119]. Human studies have shown increased serum indole-3-lactic acid levels in sedentary adults after 6 weeks of aerobic exercise training, suggesting that exercise may increase circulating levels of some microbiota-associated indole metabolites [120], whereas fecal indole-3-acetic acid levels decreased in wheel-running mice [118]. Responses to exercise therefore differ among indole derivatives, and it remains unclear whether exercise can consistently increase the levels of barrier-protective AhR ligands within the intestinal lumen.

Unlike metabolic substrates such as lactate that can be directly utilized by gut microbes, the muscle-derived endocrine signaling–intestinal epithelial repair–hypoxic niche axis primarily acts by regulating the state of the intestinal epithelium and thereby indirectly shaping the microbial colonization environment. Skeletal muscle contraction-associated signals can activate p38 MAPK and promote peroxisome proliferator-activated receptor-γ coactivator 1α (PGC-1α) gene transcription [121], while AMPK can directly phosphorylate PGC-1α and enhance its transcriptional coactivator activity [122]. In human studies, a single bout of endurance exercise increased the nuclear abundance of PGC-1α in the skeletal muscle of trained men [123]. In mouse skeletal muscle, upregulation of PGC-1α can promote fibronectin type III domain-containing protein 5 (FNDC5) expression, and proteolytic cleavage of FNDC5 releases irisin [124]. Together, these studies support an upstream molecular link involving “skeletal muscle contraction–AMPK/p38 MAPK–PGC-1α–FNDC5–irisin”; however, these individual steps have not been sequentially validated within the same exercise model.

At the intestinal level, exogenous irisin treatment can ameliorate dextran sulfate sodium (DSS)-induced colitis, accompanied by alterations in microbiota composition [125]. In mice with high-fat diet plus 2,4,6-trinitrobenzenesulfonic acid (TNBS)-induced colitis, voluntary exercise was accompanied by increased plasma irisin levels, reduced colonic injury, and decreased pro-inflammatory factors [126]. These findings, respectively derived from irisin intervention and exercise-associated changes, suggest that irisin may participate in intestinal repair following exercise. Further mechanistic studies using intestinal ischemia–reperfusion and cellular hypoxia/reoxygenation models have shown that irisin can bind to integrin αVβ5 on intestinal epithelial cells and activate the AMPK–uncoupling protein 2 (UCP2) pathway. Irisin reduces oxidative stress, mitochondrial dysfunction, and epithelial cell apoptosis, restores tight junctions, and decreases intestinal permeability. Blocking αVβ5, AMPK, or UCP2 attenuates these barrier-protective effects, supporting αVβ5–AMPK–UCP2 as an important pathway through which irisin mediates epithelial repair [127]. Studies using intestinal organoids have further shown that irisin can promote epithelial cell proliferation through Wnt/β-catenin and focal adhesion kinase (FAK) signaling and improve organoid recovery after injury [128].

Meanwhile, exercise can promote apelin expression in skeletal muscle and increase circulating apelin levels. Studies in exercising mice using intestine-specific apelin receptor (APJ) knockdown and conditional AMPKα1 knockdown models further showed that APJ–AMPK signaling participates in exercise-induced PGC-1α-related mitochondrial biogenesis and oxidative metabolic adaptation in the duodenum and supports epithelial renewal, providing relatively direct experimental support for an “exercise–apelin/APJ–AMPK–intestinal epithelial metabolic remodeling” pathway [129]. However, these exercise and epithelial-repair studies did not directly measure colonic epithelial oxygen consumption or luminal oxygen or nitrate levels under exercise conditions. Therefore, it cannot yet be concluded that exercise-associated irisin/apelin signaling remodels the hypoxic colonic niche.

Independent studies of intestinal ecology have shown that colonic epithelial oxidative metabolism and inflammatory status can influence competition between facultative and obligate anaerobes by regulating the luminal availability of oxygen and nitrate. Oxygen and nitrate can serve as respiratory electron acceptors for some facultatively anaerobic members of the Enterobacteriaceae, and increased availability of these electron acceptors favors the expansion of genera such as *Escherichia* and *Salmonella*. Conversely, reducing the availability of these electron acceptors weakens the respiratory advantage of Enterobacteriaceae and creates more favorable conditions for niche maintenance and SCFA production by obligately anaerobic fermenters such as *F. prausnitzii*, *Roseburia*, certain members of *Eubacterium*, and *Coprococcus* [48,106,110,111,112]. In particular, butyrate can activate peroxisome proliferator-activated receptor γ (PPARγ) in colonic epithelial cells, promote fatty acid β-oxidation and epithelial oxygen consumption, and suppress *Nos2*/iNOS-related nitrate generation, thereby reducing the luminal bioavailability of oxygen and nitrate, limiting Enterobacteriaceae expansion, and establishing feedback that favors the maintenance of butyrate-producing bacteria [110].

Accordingly, if exercise-associated irisin/apelin signaling produces similar epithelial metabolic repair effects in the colon, it may further influence the hypoxic, low-nitrate niche and patterns of microbial competition by restoring colonic epithelial oxidative metabolism and increasing oxygen consumption while reducing inflammation-associated nitrate generation. On this basis, the muscle-derived endocrine signaling–intestinal epithelial repair–hypoxic niche axis can be considered a candidate model linking these two mechanistic modules. Experimental evidence independently supports both the “exercise/muscle-derived signal–intestinal epithelial repair” module and the “colonic oxygen/nitrate–microbial competition” module, but a continuous mechanistic link between them has yet to be established.

These three pathways connect exercise-induced host adaptation with changes in microbial function through substrate delivery, utilization of exercise-associated metabolites, and the intestinal epithelial niche, respectively, although the completeness of the supporting evidence differs among them. The following sections focus primarily on direct studies of different exercise modalities and further discuss exercise-associated changes in the gut microbiota, their immune effects, and their tumor-related implications.

### 3.2. Effects of Endurance Exercise on the Gut Microbiota and Related Immune Effects

Under conditions of an appropriate training load, adequate recovery, and regular and sustainable participation, endurance exercise can induce changes in the composition and function of the gut microbiota, although findings are not entirely consistent across studies [130]. These differences are related to factors such as dietary patterns, energy intake, body fat levels, training protocols, study populations, and analytical methods. Some intervention studies have also observed concurrent changes in the gut microbiota, intestinal barrier, and inflammation-related markers.

In a preclinical melanoma model, endurance exercise provides a relatively complete evidence chain linking “exercise–microbial metabolism–antitumor immunity.” Endurance exercise can remodel gut microbial function, enhance folate-dependent one-carbon metabolism, and increase formate levels in cecal contents and serum. Following fecal microbiota transplantation (FMT) from endurance-trained mice into antibiotic-pretreated recipient mice, formate levels increased in the cecal contents, serum, and tumor tissues of the recipients, indicating that the exercise-remodeled microbiota had an enhanced capacity for formate production and suggesting that gut-derived formate may enter the systemic circulation and reach tumor tissues. Formate can activate nuclear factor erythroid 2-related factor 2 (Nrf2) signaling in CD8^+^ T cells, promote the Tc1-like differentiation and expansion of tumor antigen-specific CD8^+^ T cells, increase eomesodermin (Eomes) expression and IFN-γ and granzyme B (GzmB) production, and enhance antitumor effects in tumor-draining lymph nodes and the TME, thereby improving ICI efficacy [63]. However, in colorectal tumors enriched with *Fusobacterium nucleatum*, formate produced by *F. nucleatum* can promote the nuclear translocation of AhR in tumor cells, increase aldehyde dehydrogenase (ALDH) activity, enhance cancer cell stemness and invasiveness, and increase pulmonary tumor nodules in an experimental metastasis model, accompanied by an increase in Th17 cells, thereby promoting tumor progression [64]. The opposing findings of these two studies may be related to differences in the site and duration of formate exposure, the principal target cells, host immune status, and tumor stage. The antitumor effect in the melanoma model depends on Nrf2 signaling in CD8^+^ T cells, whereas the tumor-promoting effect in colorectal tumors is characterized primarily by enhanced AhR-associated stemness and invasive programs in tumor cells. Therefore, the direction of formate effects should be interpreted in the context of its mode of exposure, target cells, and host background.

With respect to the currently available exercise evidence, the mouse melanoma model has provided relatively systematic validation of a causal chain in which endurance exercise enhances CD8^+^ T-cell effector function and improves ICI efficacy through microbiota-derived formate. However, it remains to be determined which exercise-induced changes in the intestinal environment enhance microbial folate-dependent one-carbon metabolism and pyruvate formate-lyase (PFL)-mediated formate production, and whether increased PFL gene abundance is accompanied by increased transcription, enzymatic activity, and formate production flux. Studies in other colorectal cancer models have shown that treadmill training and voluntary wheel-running aerobic exercise interventions are often accompanied by changes in mucin-utilizing bacteria, microbial groups associated with SCFA production, and the metabolic functions of commensal bacteria, with these changes paralleling improvements in barrier function, reductions in inflammation, decreased tumor formation, or improved tumor phenotypes [131,132]. In addition, FMT of pre- and post-exercise samples from one selected breast cancer survivor with a favorable microbiota response into germ-free mice in a breast cancer model resulted in a trend toward reduced tumor volume and alterations in cytokine profiles [133], suggesting that exercise-associated remodeling of the gut microbiota may transmit some host responses associated with tumor control.

Human evidence primarily comprises studies examining associations with physical activity/cardiorespiratory fitness and standardized endurance-training interventions. Higher levels of physical activity or cardiorespiratory fitness are often accompanied by greater microbial richness, a higher potential for SCFA production, and enrichment of butyrate-associated microbial taxa [134,135]. Standardized endurance training can alter gut microbiota composition and SCFA-related functions in sedentary, overweight, or obese individuals, although these effects are influenced by baseline adiposity and metabolic status, and some adaptations regress after cessation of training [136,137]. Studies of single bouts of long-distance endurance exercise provide more direct evidence of rapid microbial responses to exercise-induced metabolic perturbations. The abundance of *Veillonella* increases after marathon running, and *Veillonella atypica* is capable of utilizing lactate to produce propionate [115]. After a half-marathon, fecal metabolites, including organic acids, and the composition of some microbial taxa were altered, suggesting that the gut microbial ecosystem can respond over a relatively short period to changes in the intestinal luminal environment induced by endurance exercise [138]. However, these studies did not assess endpoints related to tumor immunity or treatment responses and are therefore more appropriately regarded as translational human evidence for an “exercise metabolism–microbial functional response” link rather than as evidence from which antitumor benefits can be directly inferred.

Cross-sectional studies have shown that an active lifestyle or a higher volume of endurance training is associated with specific features of microbial composition and metabolic function [139,140]. These studies did not directly measure substrate delivery or changes in the mucus layer. In light of the gastrointestinal transit, fermentable-substrate utilization, and barrier mechanisms discussed above, regular endurance exercise may provide a more stable intestinal environment for SCFA production, barrier homeostasis, and mucosal immunity by regulating the rhythm of substrate supply, supporting maintenance of the mucus barrier, and attenuating low-grade inflammation. At the immune level, a prospective non-randomized controlled study in individuals with Lynch syndrome used a 12-month cycling intervention and observed increased peak oxygen uptake (VO_2_peak), decreased colonic and circulating inflammatory markers, and enhanced immune features associated with CD8^+^ T cells and NK cells in the colonic mucosa [141]. The study did not assess the gut microbiota and therefore supports a translational human link between “exercise–colonic mucosal immunity” but cannot be considered evidence of microbiota mediation.

In some tumor models, appropriately dosed endurance exercise is accompanied by changes in microbial metabolism, intestinal barrier function, and immune status, alongside reduced tumor progression or improved treatment responses. Among these, the one-carbon metabolism–formate–Tc1-like CD8^+^ T-cell chain in melanoma models has relatively complete evidence for microbiota mediation, whereas colorectal and breast cancer models primarily demonstrate associations or translational effects between exercise-associated microbiota changes and improvements in tumor phenotypes, with the mediating relationships remaining incomplete. Current human studies mainly support associations of physical activity, cardiorespiratory fitness, or exercise training with microbial and immunometabolic characteristics; there is currently no evidence demonstrating that changes in the gut microbiota mediate the antitumor effects of exercise.

### 3.3. Effects of High-Intensity/Strenuous Exercise on the Gut Microbiota and Related Immune Effects

High-intensity training per se should not be equated with adverse intestinal stress. Prescribed HIIT and SIT typically incorporate clearly defined intervals, total training load, and recovery periods, whereas ultra-endurance exercise, prolonged continuous strenuous exercise, or high training loads with inadequate recovery are more likely to be accompanied by intestinal hypoperfusion, heat stress, and dehydration. Therefore, the adverse effects discussed in this section primarily refer to conditions involving excessive total training load or insufficient recovery, particularly when combined with heat stress, dehydration, or low energy availability, rather than to high-intensity training in general.

Under these conditions, redistribution of splanchnic blood flow can lead to intestinal hypoperfusion, accompanied by intestinal epithelial injury and increased permeability [142,143]. Overtraining models have also demonstrated immunometabolic disturbances and reduced microbial diversity [144]. When the intestinal epithelial barrier is compromised, LPS and other microbe-associated molecules are more prone to translocation, and LPS can amplify inflammatory signaling through pathways such as TLR4–NF-κB/c-Jun N-terminal kinase (JNK) [35]. These changes do not imply that strenuous exercise necessarily promotes tumor progression. However, if barrier injury, translocation of microbial molecules, and inflammatory activation occur repeatedly or persist, they may create an inflammatory milieu unfavorable to gut microbial homeostasis and antitumor immune surveillance and may weaken the effector immune capacity required for responses to immunotherapy.

In the context of metabolic abnormalities such as obesity or insulin resistance, some studies suggest that short-term high-intensity or interval training can be accompanied by changes in microbial composition, metabolic function, and inflammation-related markers. In mice with obesity, 6 weeks of HIIT increased the α-diversity of the colonic and fecal microbiota, increased several Bacteroidales-related taxa, and enhanced the predicted functional potential of microbial metabolic pathways, including the tricarboxylic acid cycle. These changes were partly opposite in direction of those associated with a high-fat diet [145]. In individuals with insulin resistance, prediabetes, or type 2 diabetes, both SIT and MICT increased Bacteroidetes and decreased *Clostridium*. *Lachnospira* increased after SIT, whereas *Veillonella dispar* increased after MICT; tumor necrosis factor-α (TNF-α) and lipopolysaccharide-binding protein (LBP) also decreased [146]. These findings indicate that short-term SIT/MICT can be accompanied by changes in specific microbial taxa, predicted metabolic functions, and inflammatory markers, although it remains unclear whether the microbiota changes contribute to the observed metabolic and inflammatory improvements.

**Figure 3:**
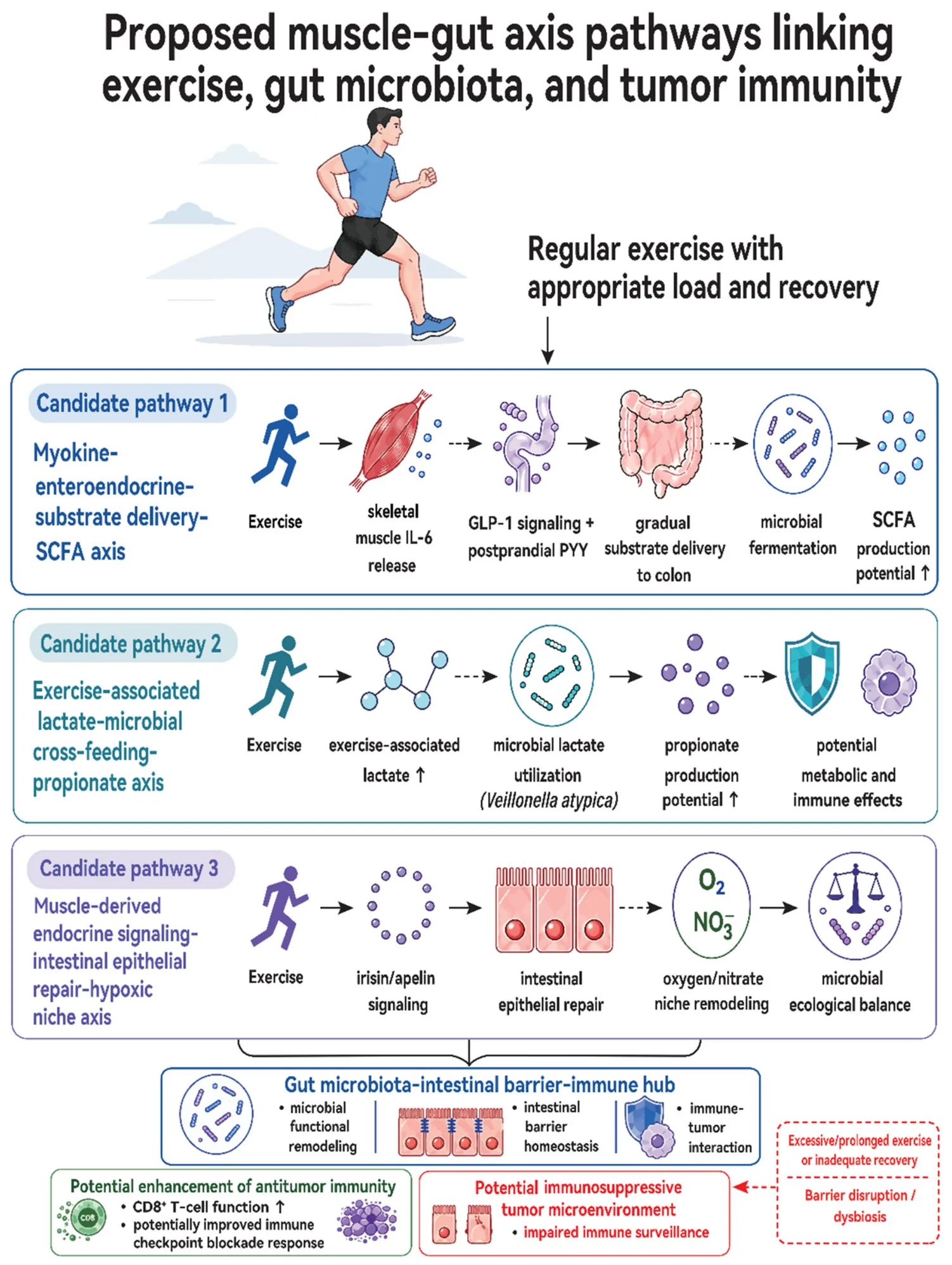
Proposed framework illustrating how exercise may regulate the gut microbiota through the muscle–gut axis and potentially influence tumor immunity and treatment responses. Three candidate pathways are summarized: the myokine–enteroendocrine–substrate delivery–SCFA axis, the exercise-associated lactate–microbial cross-feeding–propionate axis, and the muscle-derived endocrine signaling–intestinal epithelial repair–hypoxic niche axis. Appropriate exercise load and recovery may support microbial metabolic function, intestinal barrier homeostasis, and antitumor immunity, whereas excessive or prolonged exercise and inadequate recovery may contribute to barrier disruption, dysbiosis, and a potentially immunosuppressive tumor microenvironment. Solid arrows indicate component-level relationships supported within the cited experimental contexts, whereas dashed arrows indicate proposed or incompletely validated links. The framework summarizes component-level evidence and does not imply that the complete causal pathways have been established in a single tumor-bearing exercise model.

In patients with obesity, caloric restriction combined with resistance training and HIIT also resulted in improvements in body composition, metabolic markers, gut microbiota composition, and microbial metabolites [147], suggesting that HIIT may contribute to the metabolic benefits of a multicomponent intervention, although these benefits cannot be attributed entirely to HIIT itself. At the same time, other studies have shown that HIIT or longer-term concurrent exercise interventions do not significantly alter gut microbiota composition or diversity [148,149], indicating that the microbial effects of high-intensity exercise are influenced by baseline characteristics of the study population, exercise prescription design, intervention duration, and analytical methods. Therefore, in the context of cancer prevention and treatment, the use of HIIT/SIT should be individually evaluated according to treatment stage, physical capacity, nutritional status, gastrointestinal symptoms, treatment-related toxicities, inflammatory status, and recovery conditions. The priority should be to ensure that the exercise load is safe and tolerable and allows adequate recovery and, on this basis, to improve the metabolic and inflammatory milieu while maintaining intestinal barrier homeostasis. The evidence categories and strengths concerning changes in the gut microbiota and microbial metabolites under different exercise modalities, together with their tumor-related implications, are summarized in Table 2, while the integrated mechanistic framework is illustrated in Figure 3.

## 4. Summary and Perspectives

Current evidence supports associations between exercise, gut microbial metabolism, intestinal barrier homeostasis, and host immune status. This review proposes three candidate muscle–gut axes through which exercise may regulate the gut microbiota: the myokine–enteroendocrine–substrate delivery–SCFA axis, the exercise-associated lactate–microbial cross-feeding–propionate axis, and the muscle-derived endocrine signaling–intestinal epithelial repair–hypoxic niche axis. Together, these pathways suggest that exercise-associated changes in the gut microbiota may arise from altered substrate delivery, microbial utilization of exercise-associated metabolites, and remodeling of the intestinal epithelial niche, rather than simply from shifts in the abundance of individual microbial taxa. However, evidence for each axis still comes largely from individual components studied in different experimental systems, and none has been causally validated as a complete pathway in a single tumor-bearing exercise model.

Evidence that the gut microbiota mediates the antitumor effects of exercise also remains uneven. A relatively complete preclinical evidence chain comes from a mouse melanoma model, in which endurance exercise enhanced microbial folate-dependent one-carbon metabolism and formate output, with microbiota-derived formate promoting CD8^+^ T-cell antitumor activity and ICI efficacy. Other colorectal and breast cancer models have reported exercise-associated changes in the gut microbiota alongside improvements in tumor phenotypes, but most lack direct tests of microbial or metabolite mediation. Human studies remain largely associative and do not yet establish that exercise improves tumor immunity or treatment responses through the gut microbiota. Exercise load and recovery should also be considered, as excessive training loads or prolonged strenuous exercise, particularly with inadequate recovery, may impair intestinal barrier homeostasis and increase inflammatory burden.

Future studies should test the proposed pathways within the same tumor-bearing exercise model, using approaches such as microbiota depletion or transplantation, microbial strain interventions, and metabolite supplementation or blockade to establish temporal and causal relationships. Clinical studies should combine standardized exercise interventions with parallel assessment of the gut microbiota, microbial metabolites, tumor immunity, and treatment outcomes while accounting for major dietary, treatment-related, medication-related, and host factors. These studies will help determine whether gut microbiota-related measures can inform individualized exercise strategies or predict treatment responses.

## Figures and Tables

**Table 1:** Overview of the Biological Sources, Sites of Action, and Tumor-Related Effects of Representative Metabolites/Molecules.

Metabolite/Molecule	Biological Source/Evidence Scope	Primary Sites of Action/Target Cells	Key Effects	Representative Cancer Types/Models	References
SCFAs					
Acetate	Gut microbiota: *B. catenulatum*, *B. pseudolongum*; *L. reuteri* intervention accompanied by increased acetate; refs. [41,42] provide evidence of tumor acetate utilization without a defined source	CD8^+^ T cells; hepatocytes; hepatic ILC3s; tumor cells	MCT4–MAPK → CD8^+^ T-cell activation ↑; GPR43–IL-6/JAK1/STAT3 ↓; HDAC ↓ → Sox13 K30 acetylation ↑/expression ↓ → IL-17A ↓; under high-fructose conditions, O-GlcNAcylation ↑ and tumor growth ↑; ACSS2-dependent acetate utilization ↑	CRC, HCC, anti-PD-1 and metabolic-stress models	[41,42,43,44,45,46]
Propionate	Gut microbial fermentation; ref. [37] used exogenous sodium propionate intervention; ref. [38] supports a microbial source; ref. [39] concerns systemic SCFA exposure	Breast cancer/CRC tumor cells; peripheral Tregs	JAK2/STAT3 ↓, ROS–p38 MAPK ↑, G_0_/G_1_ arrest and apoptosis ↑; HECTD2 ↑ → EHMT2 degradation ↑; high circulating propionate is associated with Treg ↑ and reduced benefit from anti-CTLA-4	Breast cancer, CRC, melanoma	[37,38,39,47]
Butyrate	Gut microbial fermentation: *F. prausnitzii*, *Roseburia*, *Eubacterium*, *Coprococcus*, etc.	Colonic mucosal immune cells; CD4^+^/CD8^+^ T cells; DCs in the TME	HDAC ↓; GPR109A-related mucosal homeostasis ↑; T-cell effector function and antitumor activity ↑; in the radiotherapy context, local DC STING–type I IFN responses ↓ and radiotherapy efficacy ↓	CRC, melanoma, pancreatic cancer, radiotherapy models, etc.	[25,27,28,40,48]
Valerate	Gut microbiota: *Megasphaera massiliensis*; functional effects mainly validated by direct metabolite intervention	CD8^+^ CTLs; CAR-T cells	HDAC ↓; IFN-γ/TNF-α and cytotoxic effects ↑; antitumor activity ↑	Melanoma, pancreatic cancer, adoptive cell therapy models	[28]
Bile acids					
Primary bile acids (CDCA, TCA)	Host-derived: synthesized in the liver; the gut microbiota alters the bile acid pool by regulating primary → secondary bile acid conversion	Hepatic sinusoidal endothelial cells; CXCR6^+^ NKT cells	CXCL16 ↑; hepatic accumulation of CXCR6^+^ NKT cells and antitumor effects ↑	Primary liver cancer, liver metastasis models	[49]
DCA	Host–microbiota co-metabolism: host-derived primary bile acids are transformed by 7α-dehydroxylating bacteria such as *Clostridium scindens*	CD8^+^ T cells in CRC	PMCA activity ↑ → Ca^2+^–NFAT2 signaling ↓ → CD8^+^ T-cell effector function ↓, tumor growth ↑	CRC	[50,51]
UDCA	Host–microbiota co-metabolism: formed through gut microbial conversion of host-derived CDCA; refs. [52,53,54] mainly involve exogenous UDCA intervention	Tregs; CRC cells; colonic mucosa/macrophages	TGF-β degradation ↑ → Treg-mediated immunosuppression ↓; TGR5–YAP signaling ↓; *Akkermansia* enrichment and mucus-barrier homeostasis ↑	Multiple transplantable tumor models, CRC/CAC, immunotherapy models	[52,53,54]
Tryptophan metabolites					
I3A	Tumor-resident bacteria: ref. [55] locally produced by intratumoral *L. reuteri*; tumor cell–associated/exogenous: ref. [56] involves IL4I1-associated I3A increase and validation with exogenous I3A	CD8^+^ T cells in the TME; tumor cells	AhR-dependent Tc1/IFN-γ effects ↑, ICI efficacy ↑; AhR and c-MYC degradation ↑, MHC-I ↑, tumor immunogenicity ↑	Melanoma, lymphoma	[55,56]
IAA	Gut microbiota: *B. fragilis*, *B. thetaiotaomicron* [57], *L. reuteri* [58]; ref. [33] provides microbiota-dependent indoleacetate evidence	PDAC tumor cells/neutrophils; CXCL10–CD8^+^ T-cell axis; colonic IL-35^+^ immune cells	During combination chemotherapy, MPO/ROS-related effects ↑; CXCL10 and CD8^+^ T-cell recruitment ↑; PXR–IL-35-related anti-inflammatory effects ↑, tumorigenesis ↓	PDAC, CRC, melanoma, etc.	[33,57,58]
ILA	Gut microbiota: *L. gallinarum*, *L. plantarum*, *L. reuteri*, *B. breve*	CRC cells/organoids; DC–CD8^+^ T-cell axis; Th17 cells; colonic macrophages	Tumor-cell apoptosis ↑; DC IL-12/CD8^+^ T-cell effector function ↑; RORγt–Th17/IL-17 ↓; differentiation into homeostatic macrophages ↑	CRC, CAC	[29,30,31,32]
ICA	Gut microbiota: *L. gallinarum*	CRC cells; CD4^+^ T cells/Tregs; CD8^+^ T cells	IDO1 ↓ → Kyn ↓; antagonism of Kyn–AhR signaling → Treg differentiation/infiltration ↓; CD8^+^ T-cell effector function ↑; anti-PD-1 efficacy ↑	CRC	[59]
IPA	Gut microbiota: ref. [60] supports cooperative production by *L. johnsonii* and *C. sporogenes*; ref. [61] provides microbiota–IPA association evidence	CD8^+^ T cells/Tpex; liver	*Tcf7* super-enhancer H3K27 acetylation ↑, Tpex formation/stemness ↑, ICB efficacy ↑; in [61], decreased IPA is associated with NAFLD-HCC progression	Melanoma, breast cancer, CRC, NAFLD-HCC	[60,61]
IDA	Gut microbiota: *Peptostreptococcus anaerobius*	CRC tumor cells	AhR–ALDH1A3–FSP1 axis ↑, ferroptosis ↓, tumor progression ↑	CRC	[62]
Other metabolites/molecules					
Formate	Source is context-dependent: ref. [63] is derived from exercise-remodeled gut microbiota; ref. [64] is from CRC-associated *F. nucleatum*; ref. [65] used exogenous formate supplementation	CD8^+^ T cells; CRC tumor cells	Nrf2 ↑, Tc1-like differentiation/expansion and effector function ↑, ICI efficacy ↑; in another context, AhR ↑, cancer-cell stemness and invasiveness ↑	Melanoma, CRC	[63,64,65]
Lactate	Tumor-resident microbiota-related: ref. [66] concerns L-lactate produced by tumor-resident *L. iners*; ref. [67] shows that intratumoral *E. coli* promotes local lactate elevation; ref. [68] used exogenous lactate intervention	Tumor cells; TME macrophages; CD8^+^ T cells	Metabolic rewiring and chemoradiotherapy resistance ↑; RIG-I lactylation/M2 polarization ↑; non-acidic lactate can enhance CD8^+^ T-cell stemness and antitumor function	Cervical cancer, CRC liver metastasis, etc.	[66,67,68]
Reuterin	Gut microbiota: *L. reuteri*	CRC tumor cells	ROS/protein oxidation ↑; oxidation of key cysteine/thiol groups ↑; ribosome biogenesis/protein translation ↓; tumor growth ↓	CRC	[69]
LPS	Microbial structural molecule: a component of the outer membrane of Gram-negative bacteria, not a metabolite; can translocate after intestinal barrier disruption; ref. [35] provides evidence related to direct LPS stimulation, and ref. [36] provides TLR4 mechanistic evidence	CRC/pancreatic cancer tumor cells and TLR4-related target cells	TLR4-related inflammation ↑; VEGF-C/lymphangiogenesis ↑ or angiogenesis ↑; tumor progression ↑	CRC, pancreatic cancer	[35,36]
Inosine	Gut microbiota: *B. pseudolongum* [70]; refs. [71,72] provide evidence from direct inosine action/supplementation	T cells; tumor cells	Under costimulatory conditions, A_2_A receptor-dependent Th1/IFN-γ responses ↑; UBA6 ↓, tumor immunogenicity ↑; under glucose restriction, serves as an alternative carbon source to support CD8^+^ T-cell function	CRC, melanoma, breast cancer, etc.	[70,71,72]

**Note:** Not all entries are directly derived from the gut microbiota. Source classifications follow the original studies and include gut microbiota-derived metabolites, host–microbiota co-metabolites, tumor-resident microorganism-associated metabolites, host-derived or tumor cell-associated metabolites, and microbial structural molecules. Evidence for biological sources and tumor-related effects may derive from different studies within the same row. Exogenous supplementation or direct metabolite interventions are indicated separately. Tumor-related effects reflect the corresponding study contexts and do not imply that exercise-associated changes in the same metabolite or molecule mediate those effects. ↑ and ↓ indicate the direction of effects reported in the corresponding study context. SCFAs, short-chain fatty acids; DCs, dendritic cells; Tregs, regulatory T cells; CTLs, cytotoxic T lymphocytes; CAR-T, chimeric antigen receptor T cells; ILC3s, group 3 innate lymphoid cells; NKT, natural killer T cells; CDCA, chenodeoxycholic acid; TCA, taurocholic acid; DCA, deoxycholic acid; UDCA, ursodeoxycholic acid; I3A, indole-3-aldehyde; IAA, indole-3-acetic acid; ILA, indole-3-lactic acid; ICA, indole-3-carboxylic acid; IPA, indole-3-propionic acid; IDA, trans-3-indoleacrylic acid; LPS, lipopolysaccharide; AhR, aryl hydrocarbon receptor; ICI, immune checkpoint inhibitor; ICB, immune checkpoint blockade; TME, tumor microenvironment; PDAC, pancreatic ductal adenocarcinoma; CAC, colitis-associated cancer; HCC, hepatocellular carcinoma; CRC, colorectal cancer; NAFLD, non-alcoholic fatty liver disease; Tpex, progenitor-exhausted CD8^+^ T cells.

**Table 2:** Representative Study Characteristics, Evidence Categories, and Interpretive Boundaries for Exercise Modulation of the Gut Microbiota and Microbial Metabolites.

Study/Evidence Category	Design/Sample Size	Subjects/Cancer and Treatment	Exercise/Adherence	Diet/Medication Control	Microbiota/Metabolite Methods	Main Outcomes/Tumor Endpoints	Reference
Direct tumor model: relatively complete mediation evidence	Major sub-experiments *n* = 5–16/group; within-group randomization	Mice with melanoma; ±ICI	Treadmill 5 d/wk, ~1 h/d; animals failing to complete > 10% of sessions were excluded; separate running-wheel arm	Standard diet; ABX/FMT as mechanistic interventions	16S/PICRUSt2 + metagenomics; FMT/ABX/GF; metabolomics/formate	Yes: tumor growth ↓; Tc1-like CD8^+^ T-cell effector function ↑; ICI efficacy ↑	[63]
Direct tumor model: mediation unconfirmed	Controlled animal study; 4 groups; *n* = 6–8/group	Female mice with AOM-induced colonic tumors; ND/HFD (HFD obesity)	Treadmill 20 wk; 18 m/min, 30 min/d, 5 d/wk; completion rate NR	ND/HFD fixed	Fecal/mucosal 16S V3–V4; fecal organic acids	Yes: colonic tumor formation ↓; microbiota remodeling	[131]
Direct tumor model: mediation unconfirmed	Randomized animal study; 36 male SD rats; 6 groups, *n* = 6/group	DMH-induced CRC; no anticancer treatment	Pretraining before model induction or HIIT/MIIT during model induction; 5 d/wk, ~66 min; completion rate NR	Standard diet; medication NA	16S + untargeted metabolomics; qRT-PCR/IHC/ELISA	Yes: tumor pathology/PCNA ↓; barrier improvement, inflammation ↓	[132]
Cancer survivor FMT: single-donor translational signal	Humans *n* = 10; germ-free mice *n* = 48 (12/group)	Breast cancer survivors: prior chemotherapy/surgery, 80% radiotherapy, 50% received endocrine therapy during the study; EO771 + paclitaxel	Combined exercise 12 wk, 2 sessions/wk, 60 min; light-to-moderate intensity; attendance NR	3 d dietary records at 0/12/24 wk; mice ± oligofructose	16S V3–V4 MiSeq/QIIME2; pre-/post-exercise samples from one participant with a favorable microbiota response were selected for FMT	Humans: No; mice: Yes—post-exercise FMT showed a trend toward ↓ tumor volume	[133]
Radiation-induced intestinal injury: relatively strong microbiota-dependent evidence; not antitumor mediation evidence	Multiple animal mechanistic experiments; major phenotype ~*n* = 10/group	Main mechanistic experiment: non-tumor-bearing mice, 12 Gy whole-abdominal irradiation; separate transplantable tumor + local radiotherapy experiment	Walking 15 d; 1 m/min, 35 min/d, 6 d/wk; protocol controlled	Standard diet; ABX as a mechanistic intervention	Microbiota sequencing + untargeted metabolomics; microbiota depletion/FMT	Intestinal injury/inflammation ↓, *A. muciniphila* ↑; microbiota depletion/FMT support microbiota dependence; tumor growth unchanged after radiotherapy in the transplantable tumor model	[150]
Human CRC association	Cross-sectional analysis; *n* = 179	Stage I–IV CRC; preoperative sampling; some received neoadjuvant therapy	PA questionnaire for the 1 year before diagnosis; NA	Antibiotic use within 4 wk excluded; adjusted for neoadjuvant therapy/NSAIDs, etc.	Fecal 16S V3–V4 Illumina	No: PA/BMI associated with microbiota diversity and selected genera	[134]
Human mCRC association	Secondary analysis nested within an RCT; RCT *n* = 40; IPAQ *n* = 34; 16S *n* = 39	Unresectable stage IV mCRC; no recent anticancer treatment at baseline	PA assessed by IPAQ-SF; not an exercise intervention, NA	No antibiotics/probiotics; original RCT involved Chinese herbal medicine/placebo	Fecal 16S rRNA	Yes (OS): high PA associated with microbiota features and ↓ risk of death	[151]
Animal noncancer mechanism: lactate as a candidate mediator	Multiple-group mechanistic mouse experiments; *n* varied across sub-experiments	Mice	HIIT 6 wk; lactate treatment; MCT1/2 inhibition; completion rate NR	Experimental conditions controlled; other NR	Shotgun metagenomics; MetaPhlAn 4/HUMAnN 3.9	No: lactate partially reproduced HIIT-associated microbiota changes; MCT1/2 inhibition attenuated the changes	[116]
Human endurance intervention	Pre–post intervention; *n* = 32 (lean 18/obese 14)	Sedentary adults without cancer	Supervised endurance training 6 wk, 3 d/wk; 30 → 60 min, 60 → 75% HRR; detraining 6 wk; attendance NR	Diet controlled for 3 d before sampling	16S + fecal SCFAs	No: SCFAs ↑ in lean participants; microbiota changes were influenced by obesity and could revert	[136]
Human randomized SIT/MICT intervention	RCT; randomized *n* = 26; microbiota analysis *n* = 18	Prediabetes/T2D; noncancer	SIT or MICT, 2 wk; 6 supervised training sessions	Glucose-lowering medications discontinued before testing; statistical adjustment for metformin use; diet NR	16S + qPCR; inflammatory markers/PET	No: Bacteroidetes ↑; LBP/TNF-α ↓	[146]
Human randomized HIIT intervention	RCT; randomized *n* = 60, completed *n* = 36 (IF + HIIT 15/HIIT 11/IF 10)	Women with obesity; noncancer	HIIT 8 wk, 3 sessions/wk, 25 min; adherence NR	5:2 intermittent fasting as a design factor	16S + fecal SCFAs by gas chromatography	No: fecal acetate ↑; no significant change in microbiota composition	[148]
Human long-term supervised exercise RCT	ACTIBATE ancillary RCT; *n* = 61 (20/21/20)	Sedentary adults aged 18–25 years; noncancer	24 wk supervised aerobic + resistance exercise; electronic attendance + heart-rate monitoring	Three 24 h dietary recalls; relevant medications excluded	16S + inferred function	No: no significant change in α/β diversity; changes in a small number of taxa	[149]
CRF association	Cross-sectional; microbiota analysis *n* = 39	Healthy adults; noncancer	CRF objectively assessed by VO_2_peak; NA	Diet was similar across CRF groups and included in the analysis	High-throughput microbiota sequencing + fecal SCFAs	No: VO_2_peak associated with richness/predicted function/butyrate	[135]
Acute marathon exposure/mechanistic validation	Main human cohort *n* = 25 (runners 15/sedentary 10); additional mechanistic validation	Healthy athletes; noncancer	Boston Marathon; serial sampling before/after the race; race completed	Lifestyle records; diet not standardized	16S + shotgun; strain culture/mouse validation	No: *Veillonella atypica* ↑ after the race; component-level validation of lactate entry into the gut lumen and conversion of lactate to propionate by *V. atypica*	[115]
Acute half-marathon exposure	Single-group pre/post self-controlled study; *n* = 20	Healthy amateur half-marathon runners; noncancer	Single 21.1 km run; race completed	Similar foods provided between the two sampling points; antibiotic use within the previous 12 months excluded	16S V3–V4 + untargeted LC-MS metabolomics	No: changes in 40 fecal metabolites and some microbial taxa	[138]
Ultra-endurance load boundary	Single-group repeated measures; *n* = 9	Experienced ultramarathon athletes; noncancer	96–99 km, 38–44 h; sampling at Pre/Post/recovery 10 d	Nutritional intake recorded but not standardized; medications NR	16S rRNA	No: *F. prausnitzii* and other butyrate-producing bacteria ↓	[152]

**Note:** Exercise mode and intensity terms follow the original studies; original terminology was retained when objective intensity measures were unavailable. In the “Main Outcomes/Tumor Endpoints” column, “No” denotes no assessment of tumor occurrence, growth/progression, survival, or treatment response, whereas “Yes” denotes that at least one such tumor-related endpoint was evaluated. “Relatively complete mediation evidence” denotes evaluation of exercise, microbiota/metabolites, and tumor immunity/treatment endpoints within the same experimental framework, with causal support from FMT, microbiota depletion, germ-free models, or key metabolite interventions; the term is comparative and does not imply definitive end-to-end causal proof. “Mediation unconfirmed” denotes insufficient causal validation of microbiota/metabolite mediation. Associational studies do not establish mediation or causality. ↑ indicates increase/enhancement; ↓ indicates decrease/attenuation. NR, not reported; NA, not applicable; FMT, fecal microbiota transplantation; ABX, antibiotic treatment used for microbiota depletion; GF, germ-free; ICI, immune checkpoint inhibitor; RCT, randomized controlled trial; CRC, colorectal cancer; mCRC, metastatic colorectal cancer; PA, physical activity; CRF, cardiorespiratory fitness; HIIT, high-intensity interval training; MIIT, moderate-intensity interval training; SIT, sprint interval training; MICT, moderate-intensity continuous training; AOM, azoxymethane; DMH, 1,2-dimethylhydrazine; SD, Sprague–Dawley; ND, normal diet; HFD, high-fat diet; BMI, body mass index; NSAIDs, non-steroidal anti-inflammatory drugs; HRR, heart rate reserve; IF, intermittent fasting; T2D, type 2 diabetes; SCFAs, short-chain fatty acids; IPAQ-SF, International Physical Activity Questionnaire–Short Form; OS, overall survival; PCNA, proliferating cell nuclear antigen; MCT1/2, monocarboxylate transporters 1 and 2; LBP, lipopolysaccharide-binding protein; VO_2_peak, peak oxygen uptake; qRT-PCR, quantitative reverse-transcription polymerase chain reaction; IHC, immunohistochemistry; ELISA, enzyme-linked immunosorbent assay; qPCR, quantitative polymerase chain reaction; PET, positron emission tomography; LC-MS, liquid chromatography–mass spectrometry.

## Data Availability

No new data were created or analyzed in this study. Data sharing is not applicable to this article.
